# Discovery and validation of novel protein markers in mucosa of portal hypertensive gastropathy

**DOI:** 10.1186/s12876-021-01787-5

**Published:** 2021-05-10

**Authors:** Ying Zhu, Wen Xu, Wei Hu, Fang Wang, Yan Zhou, Jianguo Xu, Wei Gong

**Affiliations:** 1 Department of Gastroenterology, Shenzhen Hospital of Southern Medical University, Shenzhen, 518000 Guangdong China; 2 Information Management Section, Bethune International Peace Hospital, Shijiazhuang City, Hebei Province China; 3 Department of Liver Disease Center, Shenzhen Hospital of Southern Medical University, Shenzhen, 518000 Guangdong China

**Keywords:** Biomarker, Label-free quantitative mass spectrometry, Liver cirrhosis, Parallel reaction monitoring, Portal hypertension gastropathy, Proteomics

## Abstract

**Background:**

Portal hypertension induced esophageal and gastric variceal bleeding is the main cause of death among patients of decompensated liver cirrhosis. Therefore, a standardized, biomarker-based test, to make an early-stage non-invasive risk assessment of portal hypertension, is highly desirable. However, no fit-for-purpose biomarkers have yet been identified.

**Methods:**

We conducted a pilot study consisting of 5 portal hypertensive gastropathy (PHG) patients and 5 normal controls, sampling the gastric mucosa of normal controls and PHG patients before and after endoscopic cyanoacrylate injection, using label-free quantitative (LFQ) mass spectrometry, to identify potential biomarker candidates in gastric mucosa from PHG patients and normal controls. Then we further used parallel reaction monitoring (PRM) to verify the abundance of the targeted protein.

**Results:**

LFQ analyses identified 423 significantly differentially expressed proteins. 17 proteins that significantly elevated in the gastric mucosa of PHG patients were further validated using PRM.

**Conclusions:**

This is the first application of an LFQ-PRM workflow to identify and validate PHG–specific biomarkers in patient gastric mucosa samples. Our findings lay the foundation for comprehending the molecular mechanisms of PHG pathogenesis, and provide potential applications for useful biomarkers in early diagnosis and treatment.

*Trial registration and ethics approval*: Trial registration was completed (ChiCTR2000029840) on February 25, 2020. Ethics Approvals were completed on July 17, 2017 (NYSZYYEC20180003) and February 15, 2020 (NYSZYYEC20200005).

**Supplementary Information:**

The online version contains supplementary material available at 10.1186/s12876-021-01787-5.

## Background

Liver cirrhosis is the end stage of chronic liver disease characterized by fibrosis and structurally abnormal nodules. Decompensation stage cirrhosis manifests as hepatic dysfunction and portal hypertension (PH) [1]. The PH-induced esophagogastric variceal bleeding (EGVB) was the main cause of death in patients with cirrhosis [1]. Therefore, it is of great significance to understand the pathogenesis and clinical manifestations of EGVB and develop effective prevention and treatment strategies accordingly to reduce the occurrence of EGVB and its related mortality.

According to *Portal Hypertensive Bleeding in Cirrhosis: Risk Stratification, Diagnosis, and Management: 2016 Practice Guidance by the American Association for the Study of Liver Diseases* [2]*,* platelet count, ultrasound, transient elastography (TE), computed tomography (CT), or magnetic resonance imaging (MRI) are suggested as non-invasive methods to evaluate the severity of cirrhosis and PH. It also proposed that patients with compensated cirrhosis with liver stiffness < 20 kPa (determined by TE) and a platelet count > 150,000/mm^3^ were very unlikely to have high-risk varices (< 5%), and endoscopy could be safely avoided in them [3]. Patients with cirrhosis who do not meet the above criteria should undergo endoscopic examination to assess the presence of varicose veins. However, the imaging examination methods mentioned above have higher requirements on operating physicians and are subjective and restrictive to a certain extent. Currently, there is still a lack of non-invasive indicators for PH severity and objective quantitative assessment. Accordingly, there remains a clinical need for new biomarkers that achieve quantified, inexpensive, and minimally invasive testing to assess the severity of PH.

For patients with gastroesophageal varices (GOV) or EGVB, endoscopic cyanoacrylate injection eradicates varices with assistance of preoperative portal vein CT angiography and endoscopic ultrasound. However, our follow-up after 1 week of operation found that portal hypertensive gastropathy (PHG) became more severe, presenting as exacerbation of redness, point bleeding or a fine reticular pattern separating areas of raised edematous mucosa, the "mosaic pattern", which may attributes to the development of gastric capillary ectasia and collateral veins after obstruction of the esophageal varices [4]. Gastroscopy can provide an accurate PHG diagnosis and classification [4, 5]. Therefore, we sampled the gastric mucosa of the patients with compensated cirrhosis before and after endoscopic cyanoacrylate injection, and attempted to discover differentially expressed proteins (DEPs) which may appropriate for non-invasive diagnosis of PH severity.

Several studies have sought to determine disease biomarkers in gastric mucosa, with a view to the development of clinically relevant risk stratification and diagnostic tests [6, 7]. However, these studies didn’t provide sufficient resolution to differentiate the stage of PHG. The need to develop new and more effective non-invasive tests is therefore still obvious. Here, we report a pilot study applying label-free quantitative (LFQ) mass spectrometry to discover potential biomarker candidates, followed by targeted parallel reaction monitoring (PRM) to verify the PHG-related protein changes between decompensated liver cirrhosis patients and normal controls. This is the first use of LFQ-PRM for PHG biomarker discovery, and establishes the methodology required for subsequent studies to evaluate specific disease biomarkers of decompensated liver cirrhosis.

## Results

### Clinical sample information

**Fig. 1:**
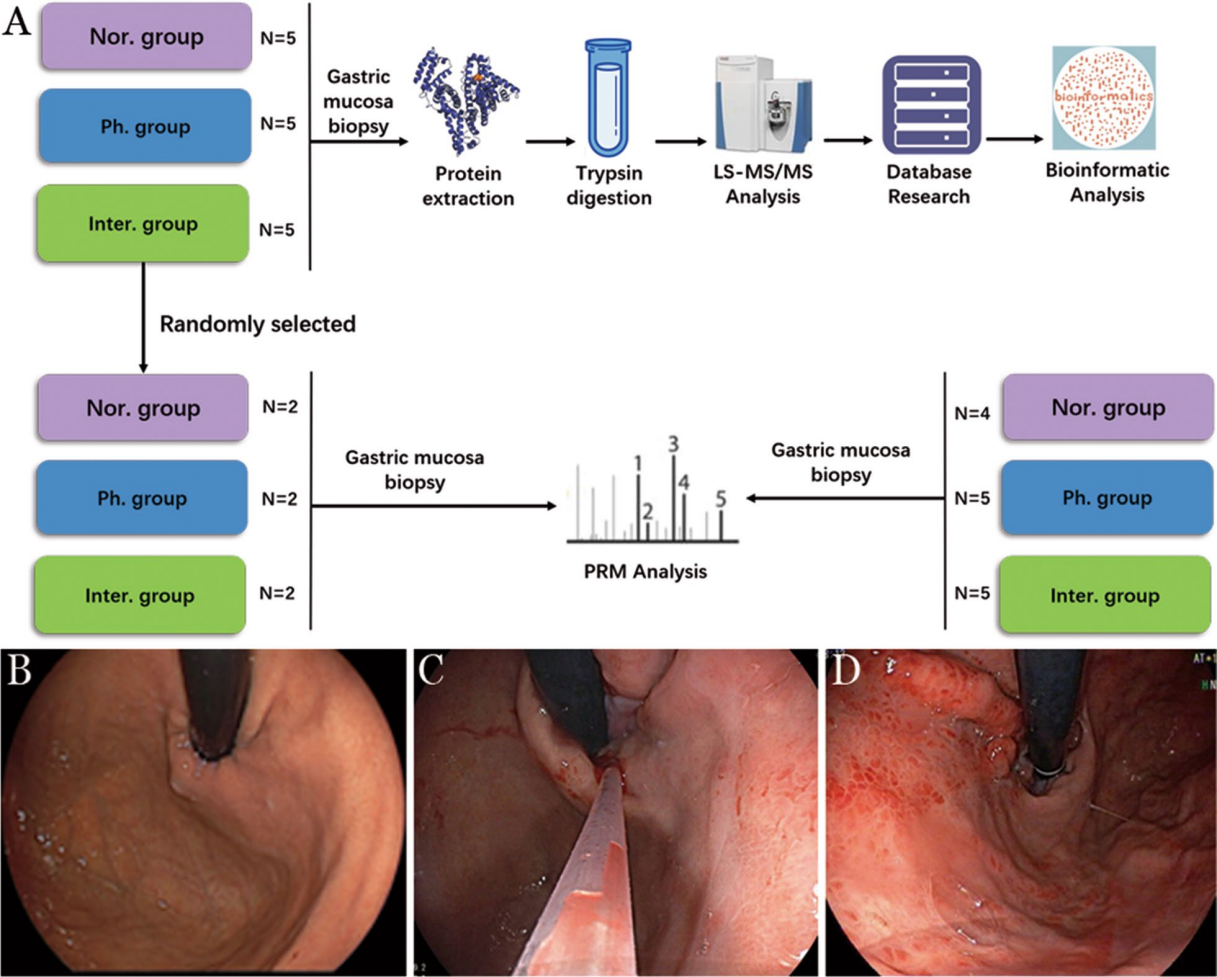
**a** Workflow describing LFQ and PRM analysis. Proteins from samples were digested by trypsin and analyzed by LS–MS/MS. The differentially expressed proteins were analyzed by database researches and validated by PRM. Nor. group = healthy individuals, Ph. group = PHG patients and Inter. group = PHG patients after endoscopic cyanoacrylate injection for 1 week. **b** The gastroscopic photographs show normal appearance of esophagus and fundus, **c** severe esophageal varices and mild PHG and **d** esophageal varices alleviation and PHG exacerbation after endoscopic cyanoacrylate injection

In this study, we evaluated the application of label-free mass spectrometry and PRM to identify and validate promising biomarkers for assessing the severity of PH in decompensated liver cirrhosis patients. The overall strategy and simplified technology roadmap are shown in Fig. 1a. In total, 29 gastric mucosa samples (n = 9 Nor group, n = 10 Ph group, n = 10 Inter group) were collected from individual patients for this study. The baseline characteristics of each group are presented in Table 1. Compared with healthy volunteers, the gastric mucosa of liver cirrhosis patients presents reddening, edematous, or even point bleeding and the appearance of the gastric mucosa after endoscopic cyanoacrylate injection were more severe (Fig. 1b–d). To provide consistency at point-of-collection, gastric mucosa samples were collected immediately during gastroscopy and processed identically.

**Table 1:** Baseline characteristics of PHG patients compared with controls

	Nor (n = 9)	Ph (n = 10)	Inter (n = 10)	*p* value
Male gender	5 (55.6%)	7 (70%)	7 (70%)	0.370
Age (years)	39.2 ± 3.32	45.8 ± 2.88	45.8 ± 2.28	0.187
Classification of PHG	0	1.4 ± 0.11	2.4 ± 0.09	< 0.05
Child–pugh score	5	8.1 ± 0.24	6.4 ± 0.15	< 0.05
Liver stiffness (kPa)	2.12 ± 0.16	32.34 ± 0.67	32.16 ± 0.67	< 0.05
Platelet count (mm^3^)	213.3 ± 12.7	62.5 ± 2.46	88.4 ± 4.39	< 0.05
Portal vein diameter (mm)	3.87 ± 1.02	15.9 ± 0.72	15.9 ± 0.72	< 0.05

Values reported as mean ± SEM. Classification of PHG: Grade 0: no PHG. Grade 1: mild reddening, mucosa was congestive but there was no mosaic pattern. Grade 2: severe redness and a fine reticular pattern separating areas of raised edematous mucosa, the "mosaic pattern," or a fine red speckling were present. Grade 3: Point bleeding was recognized in the state of grade 2.

### Identification of DEPs by LS–MS/MS Analysis

**Fig. 2:**
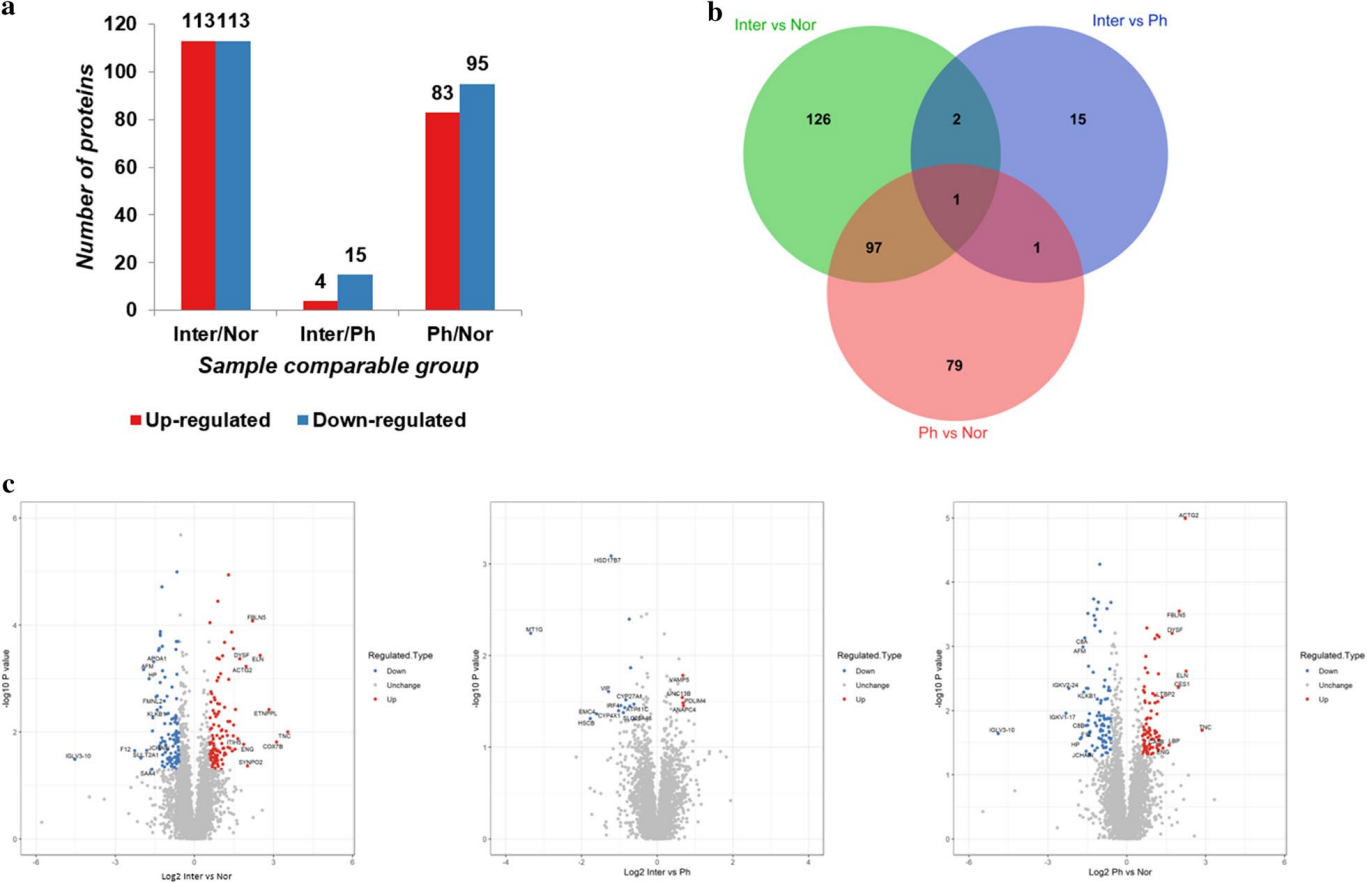
DEPs distribution in different comparative analysis groups between Inter versus Nor, Ph versus Nor and Inter versus Ph. **a** The DEPs distribution of each group is presented by bar chart. **b** A Venn diagram showing the distribution of DEPs. **c** DEPs distribution exhibits by volcano plots, which had fold change > 1.5 or < 1/1.5 and *p* < 0.05

**Fig. 3:**
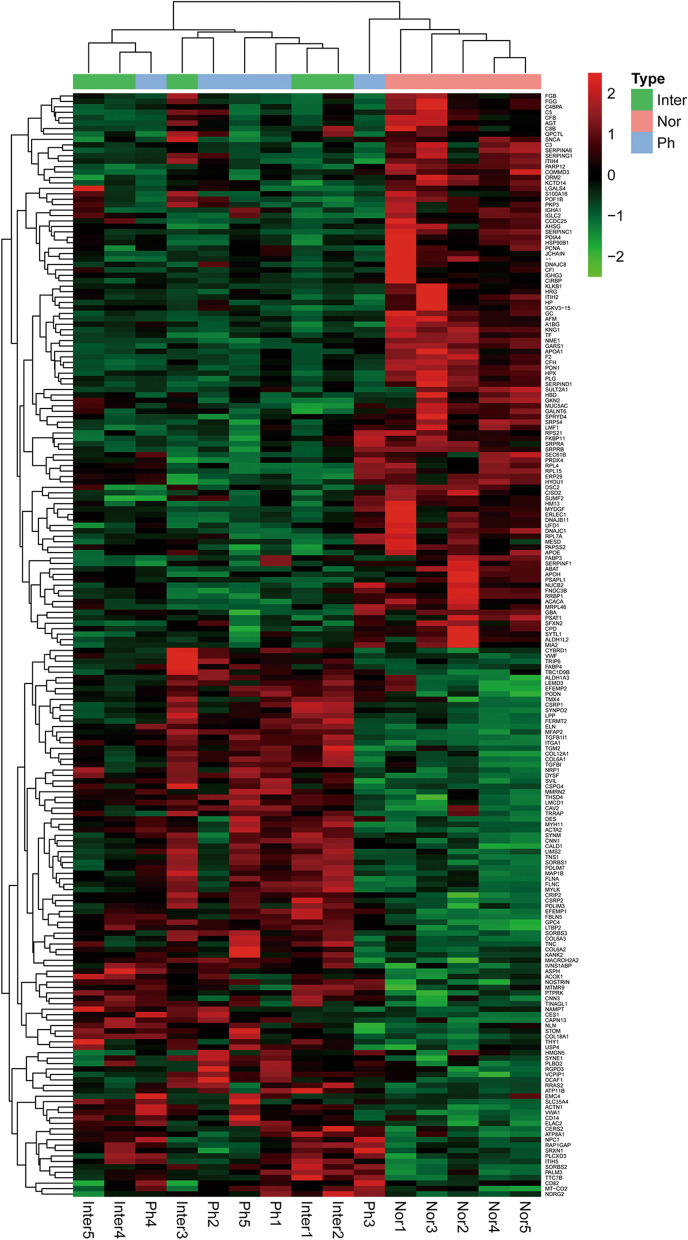
Heatmap and cluster analysis of 423 DEPs in Inter versus Nor, Ph versus Nor and Inter versus Ph. Each line in the heatmap represents the mean fold-change of up-regulation (red) or down-regulation (green) of a protein's expression levels

Using the LFQ workflow we identified a total of 6443 proteins (57,277 peptides) in the discovery cohort samples, with quantified a total of 5595 proteins. We utilized the quantitative ratio value of relative expression of protein and the corresponding *t*-test *p* value to screen the DEPs of normal controls and PHG patients before or after operation. A total of 423 proteins were found to be significantly differentially expressed under the selection criteria, of which 226 between Inter. group and Nor. group (113 up-regulated and 113 down-regulated), 19 between Inter. group and Ph. group (4 up-regulated and 15 down-regulated), 178 were found between Ph. group and Nor. group (83 up-regulated and 95 down-regulated) (Fig. 2a and Additional file 1). Out of 423 DEPs, 101 DEPs were found intersected among three group by comparison, which is shown by Venn diagram (Fig. 2b). The volcano plots based on the 423 DEPs are shown in Fig. 2c. All up-regulated and down-regulated DEPs are presented by heatmap, and their cluster analysis are performed (Fig. 3).

### Bioinformatics analysis of differentially expressed proteins

**Fig. 4:**
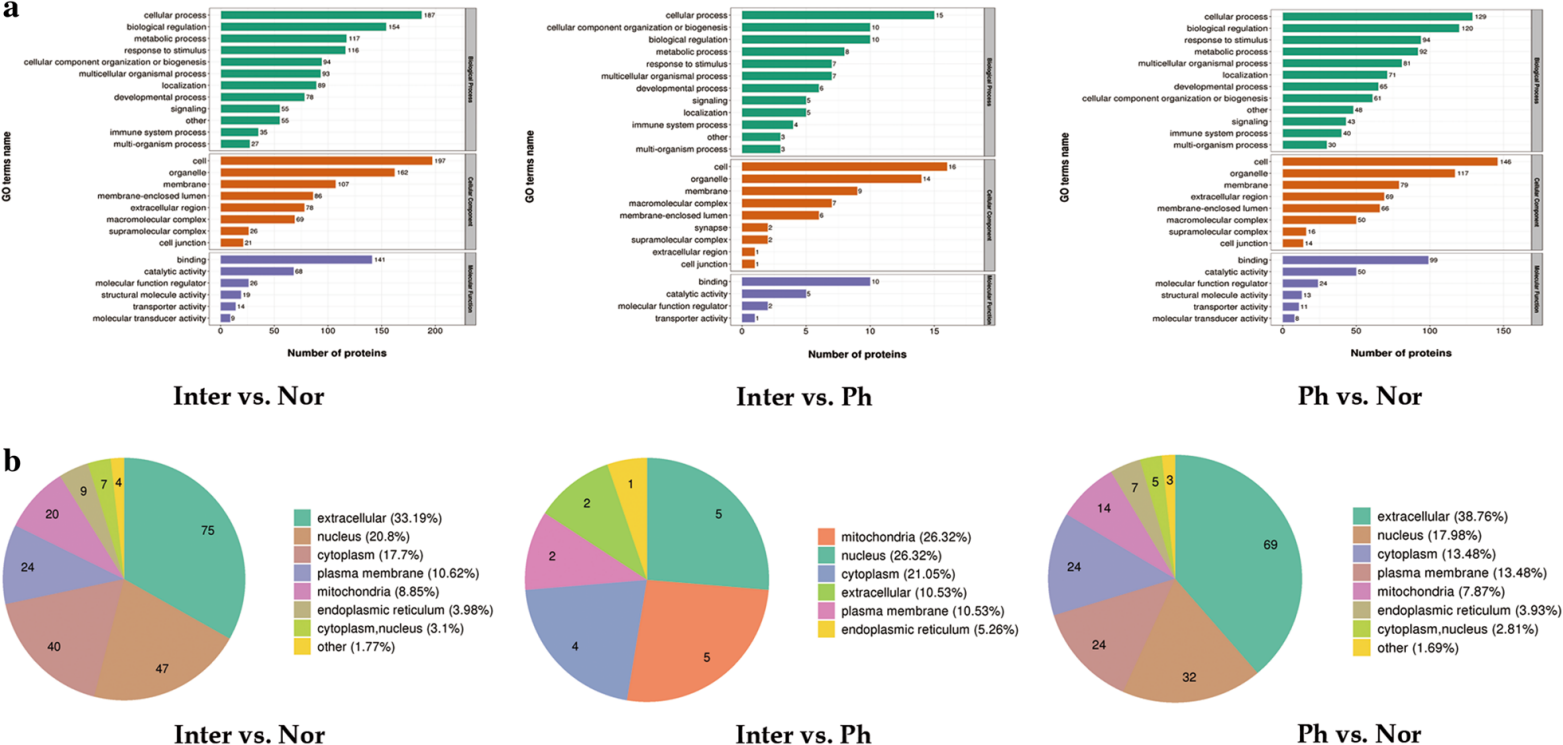
Gene Ontology (GO) level 2 analysis and subcellular location of DEPs. **a** Functional classification of DEPs for relevant biological processes, molecular functions, and cellular components. **b** Subcellular location of DEPs

Gene Ontology (GO) level 2 analysis was performed to explain relevant biological processes (BP), molecular functions (MF), and cellular components (CC) of these differentially express proteins (Fig. 4a).

In the Inter/Nor comparison, the top three biological processes associated with the 226 DEPs were: cellular process (187 proteins), biological regulation (154 proteins) and metabolic process (117 proteins). The most relevant cellular component was the cell (197 proteins), followed by organelle (162 proteins), and membrane (107 proteins). For the top three molecular functions, GO terms relating to binding (141 proteins) were the most highly represented, followed by catalytic activity (68 proteins), and molecular function regulator (26 proteins).

In the Inter /Ph comparison, the top three biological processes associated with the 19 DEPs were: cellular process (15 proteins), cellular component organization or biogenesis (10 proteins), and biological regulation (10 proteins). The most relevant cellular component was the cell (16 proteins), followed by organelle (14 proteins), and membrane (9 proteins). For the top three molecular functions, GO terms relating to binding (10 proteins) were the most highly represented, followed by catalytic activity (5 proteins), and molecular function regulator (2 proteins).

In the Ph /Nor comparison, the top three biological processes associated with the 178 DEPs were: cellular process (129 proteins), biological regulation (120 proteins), and response to stimulus (94 proteins). The most relevant cellular component was the cell (146 proteins), followed by organelle (117 proteins), and membrane (79 proteins). For the top three molecular functions, GO terms relating to binding (99 proteins) were the most highly represented, followed by catalytic activity (50 proteins), and molecular function regulator (24 proteins).

To further determine the location of DEPs, subcellular location analysis was performed (Fig. 4b). The top three subcellular locations in Inter/Nor groups were extracellular (75 proteins, 33.19%), nucleus (47 proteins, 20.8%) and cytoplasm (40 proteins, 17.7%). The top three subcellular locations in Inter/Ph groups were mitochondria (5 proteins, 26.32%), nucleus (5 proteins, 26.32%) and cytoplasm (4 proteins, 21.05%). The top three subcellular locations in Ph/Nor groups were extracellular (69 proteins, 38.76%), nucleus (32 proteins, 17.98%) and cytoplasm (24 proteins, 13.48%).

### Pathway enrichment analysis of differentially expressed proteins

**Fig. 5:**
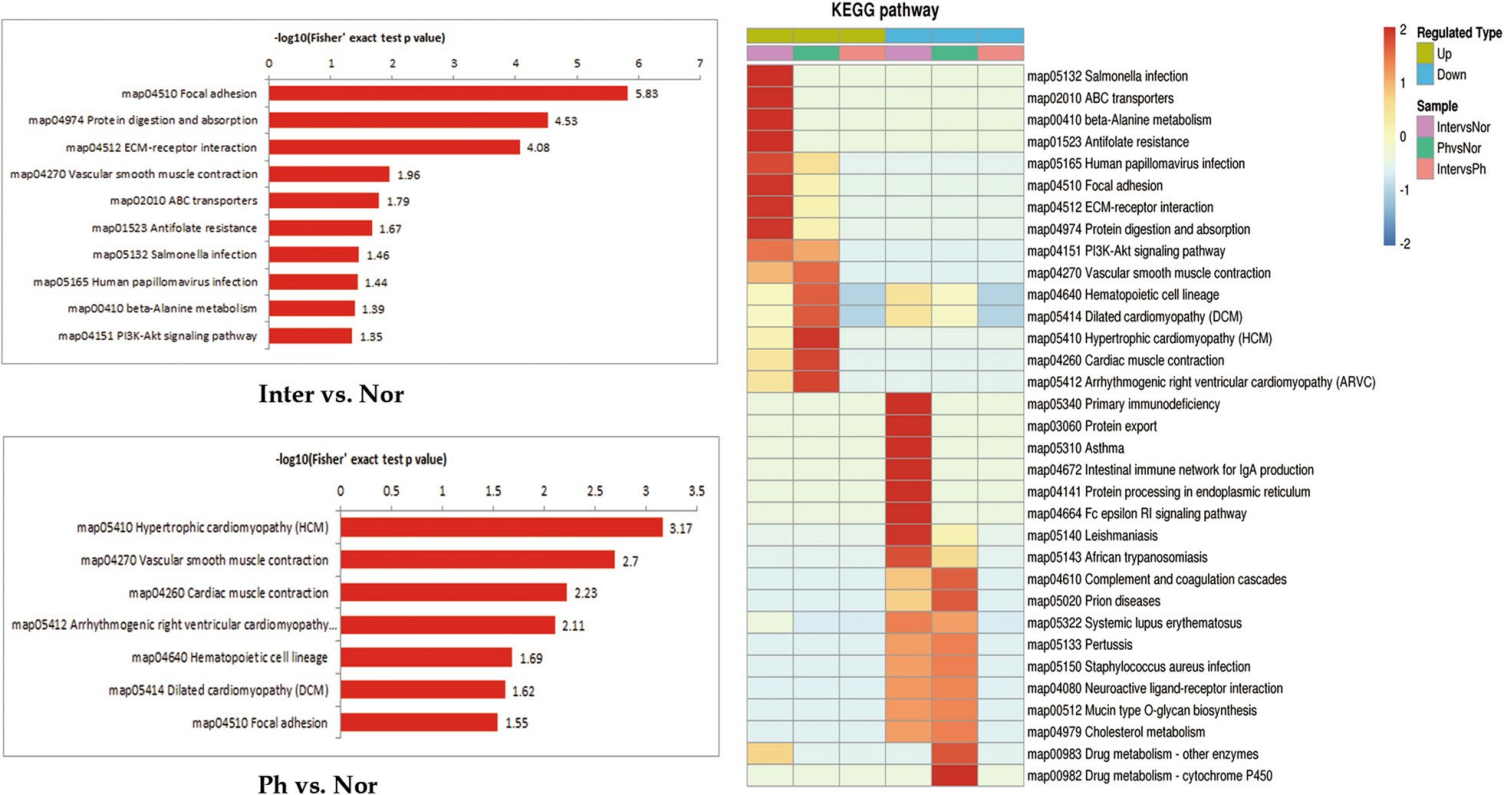
KEGG pathway analysis and heatmap [50–52] of DEPs in Inter versus Nor, Ph versus Nor and Inter versus Ph

Kyoto Encyclopedia of Genes and Genomes (KEGG) database was used as annotations for protein metabolic pathways. As it’s shown in Fig. 5, the gastric mucosa proteins up-regulated in PHG were involved in several pathways. In Ph/Nor comparison, hypertrophic cardiomyopathy (map05410), vascular smooth muscle contraction (map04270) and cardiac muscle contraction (map04260) were the top 3 enriched pathways. In Inter /Nor comparison, focal adhesion (map04510), protein digestion and absorption (map04974), and extracellular matrix (ECM)-receptor interaction (map04512) were the top 3 enriched pathways. It’s worth noting that focal adhesion (map04510) and vascular smooth muscle contraction (map04270) were both enriched in Ph/Nor groups (*p* = 0.03, *p* = 0.0001989, respectively) and Inter/Nor groups (*p* = 1.50 × 10^–6^, *p* = 0.01, respectively). However, no pathway was enriched in Inter/Ph groups.

### Protein interaction analysis (PPI)

**Fig. 6:**
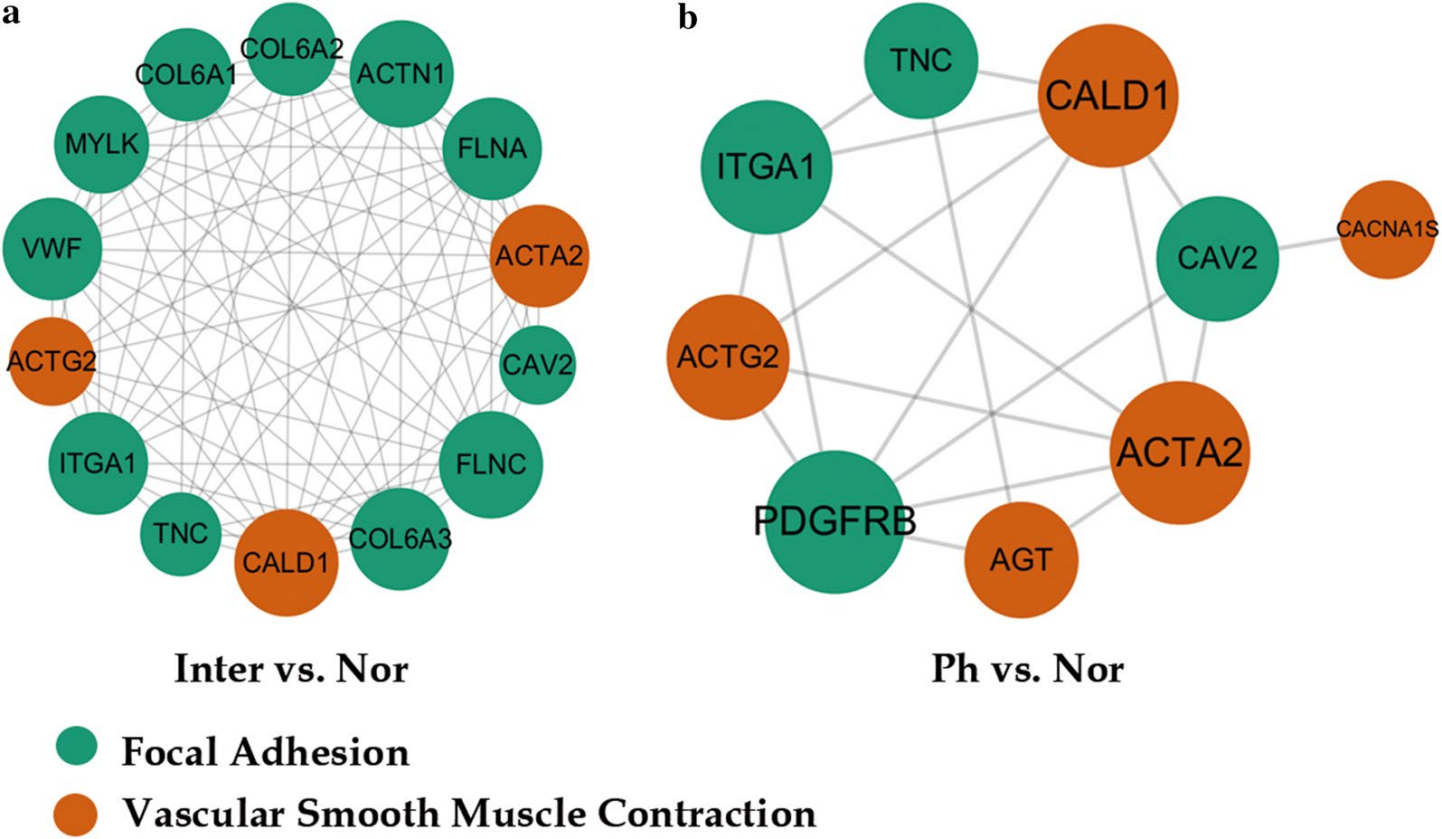
STRING protein–protein interaction (PPI) network of validated interactions between DEPs enriched in focal adhesion and vascular smooth muscle contraction. They were created using STRING v11.0 software together with a ‘Homo sapiens’ database

We used the STRING database to build a regulatory network from DEPs participated in focal adhesion and vascular smooth contraction pathway in Inter/Nor group and Ph/Nor group to visualize the predicted interactions between DEPs in PHG (Fig. 6). In Inter/Nor group, the DEPs involved in focal adhesion include COL6A1, COL6A2, COL6A3, CAV2, TNC, ITGA1, MYLK, VWF, ACTN1, FLNA and FLNC. The DEPs involved in vascular smooth contraction include MYLK, ACTA2, ACTG2 and CALD1. In Ph/Nor group, the DEPs involved in focal adhesion include PDGFRB, TNC, CAV2 and ITGA1. The DEPs involved in vascular smooth contraction include AGT, ACTA2, ACTG2, CALD1 and CACNA1S.

### PRM analysis for protein quantification

**Fig. 7:**
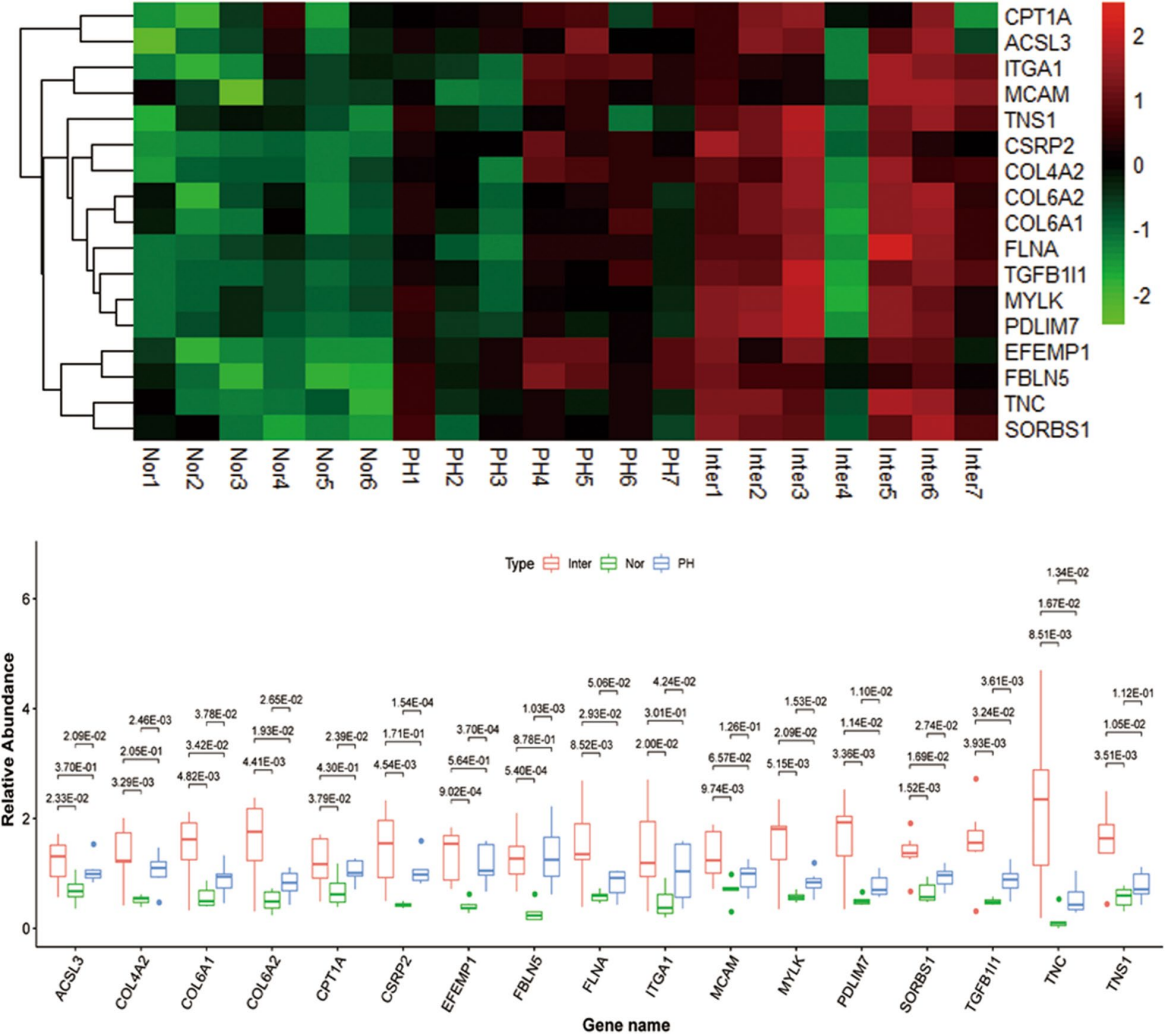
Heatmap and box plot of 17 DEPs in PHG patients before and after endoscopic cyanoacrylate injection compared with controls by PRM analysis. Each line in the heatmap represents the mean fold-change of upregulation (red) or downregulation (green) of a protein's expression levels

18 up-regulated DEPs validated by LS–MS/MS analysis related to liver diseases and gastrointestinal system were chosen for further study, their expression in gastric mucosa was measured by PRM. Due to limitation of the proteins’ properties and abundance, 17 of them were successfully quantified and 1 was not. The protein quantification results are shown in Table 2. The heatmap and boxplot are shown in Fig. 7. In accordance with LFQ, these proteins were significantly up-regulated in Ph group and Inter group compared with Nor group, the same trend was also observed in Inter group compared with Ph group.

**Table 2:** 17 differentially expressed proteins validated by parallel reaction monitoring quantification (PRM)

Protein accession	Protein gene	Inter/Nor ratio	Inter/Nor *p*-value	PH/Nor ratio	PH/Nor *p*-value	PH/Nor ratio (TMT)	Inter/PH ratio	Inter/PH *p*-value	Inter/PH ratio (TMT)
Q16527	CSRP2	3.38	4.54E−03	2.42	1.54E−04	1.99	1.40	1.71E−01	1.16
P24821	TNC	14.75	8.51E−03	3.66	1.34E−02	2.58	4.03	1.67E−02	1.30
Q15746	MYLK	2.67	5.15E−03	1.45	1.53E−02	1.16	1.84	2.09E−02	1.07
P21333	FLNA	2.64	8.52E−03	1.41	5.06E−02	1.11	1.87	2.93E−02	1.03
P50416	CPT1A	1.76	3.79E−02	1.53	2.39E−02	1.31	1.15	4.30E−01	1.11
P08572	COL4A2	2.63	3.29E−03	2.02	2.46E−03	1.60	1.31	2.05E−01	1.09
P56199	ITGA1	3.04	2.00E−02	2.19	4.24E−02	1.46	1.39	3.01E−01	1.00
P12110	COL6A2	3.24	4.41E−03	1.64	2.65E−02	1.24	1.97	1.93E−02	1.03
P12109	COL6A1	2.62	4.82E−03	1.56	3.78E−02	1.23	1.69	3.42E−02	0.88
Q9BX66	SORBS1	2.10	1.52E−03	1.42	2.74E−02	1.43	1.48	1.69E−02	1.21
O95573	ACSL3	1.76	2.33E−02	1.51	2.09E−02	1.55	1.16	3.70E−01	0.78
O43294	TGFB1I1	3.21	3.93E−03	1.78	3.61E−03	1.72	1.80	3.24E−02	1.02
P43121	MCAM	1.94	9.74E−03	1.34	1.26E−01	1.01	1.45	6.57E−02	1.26
Q9NR12	PDLIM7	3.26	3.36E−03	1.54	1.10E−02	1.23	2.11	1.14E−02	1.24
Q9HBL0	TNS1	2.80	3.51E−03	1.39	1.12E−01	1.30	2.02	1.05E−02	1.20
Q12805	EFEMP1	3.23	9.02E−04	2.90	3.70E−04	2.09	1.11	5.64E−01	1.43
Q9UBX5	FBLN5	4.56	5.40E−04	4.72	1.03E−03	2.74	0.97	8.78E−01	1.53

17 potential biomarkers were validated in 7 patients with PHG and 6 healthy individuals, using PRM

*TMT* tandem max tags

## Discussion

Currently, the severity of GOV caused by PH is critical for the prognosis of patients with decompensated liver cirrhosis. Most of the need lies in the detection of liver cirrhosis patients at risk of occurrence and the progression of PH, and early diagnosis and treatment may greatly contribute to enhancing the survival of such patients. Decompensated liver cirrhosis presents not only as GOV but also PHG. The severity of swelling and reddening of gastric mucosa [8], interstitial vascular ectasia, spindle cell proliferation and fibrohyalinosis [9] reflect the degree of PHG and are associated with severity of liver disease. Monitoring changes in the gastric mucosa proteome may have an advantage in the diagnosis of decompensated liver cirrhosis. However, targeted proteomics, which identifies promising biomarkers of disease activity and organ involvement, is rarely involved in non-invasive method for detection and diagnosis of liver cirrhosis induced PH. Our group devoted to the research of proteomics in liver disease [10, 11]. To the best of our knowledge, no proteomic analysis of gastric mucosa has been reported for the discovery of biomarkers and to describe the involvement of related biological pathways in liver cirrhosis induced PH. In this study, we performed a comparative analysis of the gastric mucosa proteome to obtain insights into the quantitative assessment and prognosis in liver cirrhosis patients via LC–MS/MS and PRM. As a result, we found that 423 proteins were differentially expressed in gastric from PHG patients before and after endoscopic cyanoacrylate injection compared to that from normal controls. Due to the importance of pathogenesis and early diagnosis of PHG, we studied the characterization and function of some differentially expressed proteins in depth.

According to KEGG function classification analysis, several pathways have been proved to be enriched in Ph/Nor and Inter/Nor group, but only focal adhesion pathway and vascular smooth muscle contraction pathway are enriched in both Ph/Nor and Inter/Nor group while others are not, indicating these two pathways may play critical roles in the process of PHG deterioration. In the process of liver fibrogenesis, focal adhesion connects hepatic stellate cells (HSCs) and ECM, and provides a direct sensor to the integrity of interaction with the extracellular environment, causing cell adhesion and migration [12, 13]. The patients with liver cirrhosis exhibit a progression of vascular smooth muscle contraction dysfunction, which presents as an increased intrahepatic vasoconstriction and reduced systemic vascular resistance [14]. The progress of PHG is related to hemodynamic alterations in the gastric mucosa, presenting as hemangiectasis [15], abnormal formation of arteriovenous shunts and blood flow augmentation [16].

Among these DEPs, PDLIM4 is the only protein which alters among the three groups, indicating cytoskeleton organization and stress fiber formation [17] may relate to development of PHG. Moreover, 17 DEPs about liver diseases and gastrointestinal system were further selected and validated by PRM based on our large-scale LS–MS/MS study. LS–MS/MS quantification is an agreeing approach to provide thorough and relative fold changes of thousands of peptides across numerous samples and to discover differential expressed peptides, based on so-called data-dependent acquisition. However, as a trade-off to the in-depth analysis, the obtained fold changes can be underestimated [18]. There DEPs are up-regulated in PHG patients and even more after endoscopic cyanoacrylate injection, indicating their expression level is consistent with severity of PHG. Among these DEPs, MYLK, TNC, ITGA1, FLNA, COL6A1, COL6A2 and COL6A3 are enriched in focal adhesion pathway. MYLK participate in smooth muscle contraction pathway at the same time. Activation of MYLK triggers gastrointestinal smooth muscle contraction with an increase of intracellular Ca^2+^ [Ca^2+^]_I_. Compared with antrum muscles, fundus muscles have a higher level of MYLK expression [19], which may contribute to the mechanism of gastric motility. In addition, MYLK also mediates cell–cell and cell-matric adhesion [20]. TNC can promote arterial smooth muscle cells proliferation and ECM elements deposition after stimulation of inflammatory cytokines and growth factors in cerebral arteries [21] and pulmonary arteries [22] by enhancing focal adhesion kinase phosphorylation [23]. These mechanisms may contribute to TNC mediated vascular remodeling [24, 25] and participate in the pathogenesis of subarachnoid hemorrhage and pulmonary arterial hypertension. FLNA couples cell cytoskeleton to ECM and regulates integrin alpha-1 (ITGA1), thus affects the function of collagen and ECM remodeling [26–29]. With the development of liver fibrosis, FLNA can be activated in HSCs and may serve as a biomarker [30]. Integrin modulates angiogenesis, regulates inflammation, and translates biliary injury into stimulation of matrix-producing mesenchymal cells in the process of fibrosis development [31]. Thereinto, ITGA1 is a receptor for laminin and collagen, it is involved in cell adhesion and correlates with inflammatory activity and fibrosis [32]. Collagen IV contributes to ECM structure and expresses extracellularly. It is structured by a heterotrimer of the alpha1(VI), alpha2(VI), and alpha3(VI) chains which are encoded by COL6A1, COL6A2, COL6A3, respectively [33].

Other validated proteins, though not enriched in the two pathways mentioned above, also play a role in the process of liver and gastrointestinal diseases. CPT1A and ACSL3, which are responsible for fatty acid oxidation, have been proved to stimulate gastric cancer and hepatocellular carcinoma cell growth and migration [34–36]. MCAM (CD146) involves in actin cytoskeleton rearrangement, blood vessel endothelial cells remodeling and intercellular junction [37]. CSRP2 expresses specifically in HSCs in liver and mediates the transdifferentiation of HSCs into myofibroblasts with the activation of TGF-β [38]. Focal adhesion protein TGFB1I1 (also known as Hic5) is induced by TGF-βand regulates myofibroblast differentiation in liver [39]. TNS1 is also localized to focal adhesions and acts as a bridge linking ECM and the actin cytoskeleton [40]. EFEMP1 and FBLN5 are ECM proteins and highly express in portal fibroblasts, they have been proved to play a role in progressive liver fibrosis [41, 42]. SORBS1 involves in cytoskeleton organization and insulin signaling pathway in human hepatoma cell line [43]. PDLIM7 is composed of PDZ and LIM7 domain, the LIM7 domain is associated with progression of liver fibrosis in hepatitis C virus infected patients [44]. Although the association with PHG is not quite clear, this study identified these DEPs as potential biomarkers on gastric mucosa for liver cirrhosis induced PH. However, more studies are required to make sure their availability as predictive biomarkers and diagnostic targets.

## Conclusion

In this study, we identified quantitative differences in expression of several proteins on gastric mucosa for PHG patients. This proteomic study demonstrates that a LFQ analysis can be useful in detection of novel predictive biomarkers, and 17 promising proteins are further validated by PRM analysis. Our findings will provide a prospect for non-invasive early diagnosis and prediction of severity of liver cirrhosis induced portal hypertension, but more mechanisms of the interaction of these DEPs and their effect on the progression of PHG require further research.

## Methods

### Clinical samples

From March 2020 to June 2020, decompensated liver cirrhosis patients meeting the criteria below were admitted to the Department of Digestive Medicine, Shenzhen Hospital of Southern Medical University. Inclusion criteria included: (1) an age range of 15–85 years, (2) computed tomography (CT), laboratory examination and endoscopy confirmed the diagnosis of PHG according to *Portal Hypertensive Bleeding in Cirrhosis: Risk Stratification, Diagnosis, and Management: 2016 Practice Guidance by the American Association for the Study of Liver Diseases*, (3) Moderate to severe esophageal varices have no bleeding, but there is an obvious risk of bleeding, endoscopic primary prevention treatment is needed, and (4) Cases with a history of esophageal varices rupture and bleeding and secondary endoscopic prophylaxis. Exclusion criteria included acute EGVB, presinus and retrosinus portal hypertension portal hypertension, esophageal or gastric submucosal tumor, hypertrophic gastritis, and patients who have endoscopic examination contraindications. All individuals were excluded helicobacter pylori infection by Carbon-13 urea breath test or histologic examination of gastric mucosa. 5 healthy individuals who underwent physical examinations and participated in another research of our group [5] with no history of alcoholic or viral hepatitis and helicobacter pylori infection were assigned to the normal control group (Nor group), 5 liver cirrhosis patients with gastroscopy confirmed PHG were assigned to PHG group (Ph group). Ph group patients underwent gastroscopy reexamination 1 week after endoscopic cyanoacrylate injection (Inter group). Gastric mucosa samples were collected during gastroscopy by a biopsy forceps from the middle part of greater curvature by gastroscopic biopsy in Department of Endoscopy, Shenzhen Hospital of Southern Medical University. All samples were assigned to LFQ analysis, 2 individuals of each group were randomly selected to participate in PRM verification. Besides, 4 healthy individuals and 5 liver cirrhosis patients meeting the criteria above were assigned to PRM analysis. The study design was approved by the Shenzhen Hospital of Southern Medical University Ethic Committee complying with *Declaration of Helsinki*, *Operational Guideline for the Ethic Review of Biomedical Research involving Human Subject* [(2016) No.11] and *International Ethical Guidelines for Biomedical Research Involving Human Subjects, CIOMS.* All participants have signed the written informed consent.

### Protein extraction and LS–MS/MS analysis

Proteins extraction and LS–MS/MS analysis were performed according to the method of Zheng and Xie et al. [45, 46]. Gastric mucosa samples were grinded by liquid nitrogen into cell powder and then transferred to a 5-mL centrifuge tube. After that, four volumes of lysis buffer (8 M urea, 1% Protease Inhibitor Cocktail) were added to the cell powder, followed by sonication three times on ice using a high intensity ultrasonic processor (Scientz). (Note: For PTM experiments, inhibitors were also added to the lysis buffer, e.g. 3 μM TSA and 50 mM NAM for acetylation.) The remaining debris was removed by centrifugation at 12,000 g at 4 °C for 10 min. Finally, the supernatant was collected and the protein concentration was determined with BCA kit according to the manufacturer’s instructions.

For digestion, the protein solution was reduced with 5 mM dithiothreitol for 30 min at 56 °C and alkylated with 11 mM iodoacetamide for 15 min at room temperature in darkness. The protein sample was then diluted by adding 100 mM TEAB to urea concentration less than 2 M. Finally, trypsin was added at 1:50 trypsin-to-protein mass ratio for the first digestion overnight and 1:100 trypsin-to-protein mass ratio for a second 4 h-digestion.

The tryptic peptides were dissolved in solvent A (0.1% formic acid, 2% acetonitrile in water), directly loaded onto a home-made reversed-phase analytical column ( ReproSil-Pur Basic C18 column(1.9 μm, 100 μm i.d., 25 cm)). Peptides were separated with a gradient from 4 to 22% solvent B (0.1% formic acid in acetonitrile) over 70 min, 22–30% in 14 min and climbing to 80% in 3 min then holding at 80% for the last 3 min, all at a constant flow rate of 450 nL/min on a nanoElute UHPLC system (Bruker Daltonics).

The peptides were subjected to Capillary source followed by the timsTOF Pro (Bruker Daltonics) mass spectrometry. The electrospray voltage applied was 1.75 kV. Precursors and fragments were analyzed at the TOF detector, with a MS/MS scan range from 100 to 1700 m/z. The timsTOF Pro was operated in parallel accumulation serial fragmentation (PASEF) mode. Precursors with charge states 0 to 5 were selected for fragmentation, and 10 PASEF-MS/MS scans were acquired per cycle. The dynamic exclusion was set to 30 s.

### Database search

The resulting MS/MS data were processed using MaxQuant search engine (v.1.6.6.0). Tandem mass spectra were searched against the human uniprot database [Homo_sapiens_9606_SP_20200509 (20,366 entries)] concatenated with reverse decoy database. Trypsin/P was specified as cleavage enzyme allowing up to 2 missing cleavages. The mass tolerance for precursor ions was set as 20 ppm in the first search and 20 ppm in Main search, and the mass tolerance for fragment ions was set as 20 ppm. Carbamidomethyl on Cys was specified as fixed modification, and acetylation on protein N-terminal and oxidation on Met were specified as variable modifications. FDR was adjusted to < 1% and minimum score for modified peptides was set > 40.

### Bioinformatics analyses

#### Differentially expressed protein analysis

In this study, the quantitative values of each sample in three replicates were obtained by LFQ intensity. For each protein, LFQ intensity was normalized by mean of all samples. And then normalized LFQ intensity were taken as log2 transform (so that the data conforms to the normal distribution), and *p* value was calculated by the two-sample two-tailed T-test method. When *p* value < 0.05 and protein ratio > 1.5 was regarded as up-regulation. When *p* value < 0.05 and protein ratio < 1/1.5 was regarded as down-regulation.

#### GO classification [47]

Gene Ontology (GO) annotation proteome was derived from the UniProt-GOA database (www. http://www.ebi.ac.uk/GOA/). Firstly, DEPs were mapped to GO IDs by protein accession. If some DEPs were not annotated by UniProt-GOA database, the InterProScan soft would be used to annotated protein’s GO functional based on the protein sequence alignment method. Then DEPs were classified by Gene Ontology annotation based on three categories: biological process, cellular component and molecular function. A bar plot graph was used to present GO terms by visualization R package “ggplot2” in RStudio.

#### KEGG pathway enrichment [48]

KEGG database was used pathways. Firstly, using KEGG online service tools KAAS to annotated protein’s KEGG database description. Then mapping the annotation result on the KEGG pathway database using KEGG online service tools KEGG mapper. DEPs enriched pathways were identified by a two-tailed Fisher’s exact test. The pathway with *p* value < 0.05 was considered significant. A bubble plot graph was used to present enriched pathway by visualization R package “ggplot2”. All calculation and visualization steps were performed in RStudio.

#### Protein–protein interaction network

All DEPs accessions were searched against the STRING database version 11.0 for protein–protein interactions. Only interactions between the proteins belonging to the searched data set were selected, thereby excluding external candidates. STRING defines a metric called “confidence score” to define interaction confidence; we fetched all interactions that had a confidence score > 0.7 (high confidence). Interaction network form STRING was visualized in CytoScape software.

### Parallel reaction monitoring (PRM)

We use PRM analysis to verify protein expression levels according to the method of Zhang et al. [49]. The resulting MS data were processed using Skyline (v.4.1.0.18166). Peptide settings: enzyme was set as Trypsin [KR/P], Max missed cleavage set as 0. The peptide length was set as 7–25, Variable modification was set as Carbamidomethyl on Cys and oxidation on Met, and max variable modifications was set as 3. Transition settings: precursor charges were set as 2, 3, ion charges were set as 1, ion types were set as b, y, p. The product ions were set as from ion 3 to last ion, the ion match tolerance was set as 0.02 Da.

### Statistics

All data were processed using Microsoft Excel 2019 (Microsoft Corporation, Redmond, WA, USA). Data were expressed as the mean ± SEM, and comparisons of differences between two groups were analyzed using a one-way analysis of variance (ANOVA). Count data are expressed as a ratio, and the χ^2^ test was used for comparisons of differences between groups. *p* < 0.05 was designated to be of statistical significance. Statistical tests were conducted using SPSS v26.0 (SPSS, Chicago, IL, USA).

## Supplementary Information

**Additional file 1.** 423 differentially expressed proteins (DEPs) between normal controls and PHG patients before or after operation.

## Data Availability

The datasets supporting the conclusions of this article are included within the article and its additional files.

## Abbreviations

BP: Biological processes
CC: Cellular components
CT: Computed tomography
DEPs: Differentially expressed proteins
EGVB: Esophagogastric variceal bleeding
ECM: Extracellular matrix
GO: Gene ontology
GOV: Gastroesophageal varices
HSCs: Hepatic stellate cells
KEGG: Kyoto Encyclopedia of Genes and Genomes
LFQ: Label-free quantitative
MF: Molecular functions
MRI: Magnetic resonance imaging
PH: Portal hypertension
PHG: Portal hypertensive gastropathy
PPI: Protein–protein interaction
PRM: Parallel reaction monitoring
TE: Transient elastography
